# Barriers to disease notification among primary healthcare physicians in West Bank, Palestine: a cross-sectional survey

**DOI:** 10.1186/s12913-026-15297-1

**Published:** 2026-08-10

**Authors:** Ayah Tuffaha, Emily White Johansson, Subhi Alshaer, Samer Asad

**Affiliations:** 1 Hebron Preventive Medicine Department, Palestinian Ministry of Health, Hebron, Palestine; 2 Mediterranean and Black Sea Programme in Intervention Epidemiology Training (MediPIET), European Centre for Disease Prevention and Control (ECDC), Stockholm, Sweden; 3 Department of Women’s and Children’s Health, Global Health and Migration Unit, Uppsala University, Uppsala, Sweden; 4 Department of Global Public Health, Karolinska Institutet, Stockholm, Sweden; 5 Jericho Preventive Medicine Department, Palestinian Ministry of Health, Jericho, Palestine; 6 Palestinian Ministry of Health, Ramallah, Palestine

**Keywords:** Primary healthcare, Infectious disease notification, Knowledge, attitude, and practice, West bank, Palestine, Public health surveillance

## Abstract

**Introduction:**

Underreporting remains a persistent challenge for infectious disease surveillance systems. Evidence on notification knowledge and practices among physicians in the West Bank remains limited, although they play a critical role in disease notification. We aimed to assess the knowledge, attitudes, and practices of primary healthcare physicians in disease notification, identify factors associated with appropriate notification of suspected mumps, measles, and chickenpox cases, and describe barriers to notification.

**Methods:**

We conducted a cross-sectional survey on primary healthcare physicians in the Palestinian Ministry of Health primary healthcare centers between August 2024 and May 2025. The questionnaire assessed knowledge, attitudes, and self-reported notification practices. Multivariable logistic regression was used to identify factors associated with recent notification practices.

**Results:**

Among 223 respondents, 30.9% were categorized as having a good knowledge score of the disease notification process across multiple operational domains of disease notification, while 93.3% were categorized as having a good attitude score. Common misconceptions included laboratory confirmation being required for notification (61.0%) and that surveillance case definitions require laboratory criteria (65.5%). Over 80% of respondents notified their last-encountered measles, mumps, or chickenpox case. However, timely notification was lower; 52.4% of measles cases were reported immediately, compared with 19.3% of mumps and 14.0% of chickenpox cases reported within the recommended timeframes. Commonly reported barriers included lack of time (60.5%), and uncertainty about which diseases to notify (38.1%) and when (32.3%). Knowledge and attitude scores were not significantly associated with notification practices, although limited observations and composite scores may have obscured these associations. Physicians who received recent supervisory feedback on disease notification had higher odds of notifying chickenpox cases (OR: 4.01, 95%CI: 1.40-11.43). Compared to general practitioners, other medical specialties had lower odds of notifying mumps (OR: 0.21, 95%CI: 0.06–0.81) and chickenpox cases (OR: 0.23, 95%CI: 0.08–0.69).

**Conclusions:**

Despite positive attitudes towards the notification system, knowledge gaps and misconceptions may contribute to underreporting of notifiable conditions. Strengthening training, clarifying guidance, improving feedback mechanisms, and addressing identified barriers and preferences may enhance disease notification performance by primary healthcare physicians in the West Bank.

**Supplementary Information:**

The online version contains supplementary material available at https://doi.org/10.1186/s12913-026-15297-1.

## Introduction

The global community has faced numerous epidemics and infectious disease outbreaks in recent years, notably the COVID-19 pandemic that caused over seven million deaths worldwide [1]. Such events have underscored the critical importance of strong public health surveillance systems to detect and respond to outbreaks in a timely manner with the goal of preventing excess morbidity and mortality in a population.

Primary healthcare centers play an essential role in preventing and containing outbreaks through the early notification and management of patients who present with infectious conditions that could spread to others [2, 3]. National public health departments establish their own regulations regarding communicable disease notification and the nature and timing of the notification process [3, 4]. It is crucial that primary healthcare workers have a good understanding of these guidelines and are capable of timely notifications to maintain a functional infectious disease surveillance system.

The Palestinian Ministry of Health operates approximately 76% of primary healthcare centers in the West Bank, and there were over two million visits to both general and specialized primary healthcare physicians recorded in 2024 [5]. Primary healthcare physicians, therefore, play an essential role in the detection and notification of cases to the Palestinian Preventive Medicine departments. However, the current surveillance system in Palestine has been described as weak, fragmented, and with inconsistent protocols and insufficient data collection [6]. The passive detection system further contributes to an increased gap in surveillance data due to underreporting [6].

There is currently limited understanding of primary healthcare physicians’ perceptions of the notification system in the West Bank and any barriers they have encountered in disease notification to the Palestinian Preventive Medicine departments. Various factors might influence the decision-making process of physicians in terms of disease notification including an understanding of notification timing or methods, disease relevance, and other logistical challenges or systemic barriers [7–11].

In the current study, we aimed to assess the knowledge, attitudes and practices (KAP) of disease notification among primary healthcare physicians in the West Bank, and to analyze the association between knowledge and attitude scores, physician characteristics and appropriate notification of suspected mumps, measles, and chickenpox cases to authorities. As a secondary objective, we aimed to identify perceived barriers encountered by physicians during the notification process and describe their recommendations to improve the system.

## Materials and methods

### Study design and setting

We conducted a cross-sectional survey of physicians actively working in the Palestinian Ministry of Health primary healthcare centers between 5 August 2024 and 24 May 2025. The primary healthcare centers are distributed across 14 West Bank district directorates that are divided into North (Qalqilya, Jenin, Tubas, Nablus, Tulkarm, Salfit), Middle (Ramallah, Jerusalem, Jericho) and South (Dura, Yatta, Hebron, North Hebron, Bethlehem) regions.

### Study population

The study population included physicians who were actively working in the primary healthcare centers in these districts during the study period and who were not in administrative roles (*n* = 487). The complete list of physicians in these districts and their contact information was obtained from the Palestinian Ministry of Health administration.

### Sampling and sample size

We used simple random sampling to select physicians for inclusion in the study. The sample size was calculated using OpenEpi and assumed a 50% frequency of physicians notifying at least one case of mumps, measles, or chickenpox in the past 12 months (outcome), a confidence level of 95%, and a design effect of one. Based on these calculations, the required sample size for the survey was 215 physicians, which was then adjusted up to 269 physicians based on an expected 80% response rate.

### Data collection

Data were collected using an online self-administered questionnaire in English that was created using KoboCollect, which is the standard language of medical education and clinical documentation among physicians in Palestine. Each sampled physician was contacted by phone and text message to request participation, and upon a positive response, they were sent the questionnaire for completion. Those who did not respond were contacted a second time before study exclusion.

The questionnaire consisted of 32 questions across five main sections (see Tables 1, 2 and 3 for a list of variables). The first section focused on sociodemographic characteristics of respondents. The second section assessed the respondent knowledge of the Palestinian Ministry of Health notification policies and included case scenarios related to the process, timing, and notification requirements for multiple notifiable diseases. The third section evaluated respondent perceptions and attitudes of the notification system using a series of five Likert-scale questions. The fourth section assessed respondent notification practices and behaviors in their most recent clinical encounter with a notifiable condition in the past 12 months, which included questions on how and where they notified the disease, and any barriers encountered at that time. In the fifth section, respondents could provide feedback to improve the notification process (see Table 5).

The questionnaire was developed in consultation with public health specialists, epidemiologists, and preventive medicine doctors who evaluated its content for relevance, suitability, question comprehension, and flow prior to its use in the research. A pilot study was conducted among 12 primary healthcare physicians in July-August 2024 to assess question clarity, comprehension, and the feasibility of using KoboCollect for data collection.

### Variable definitions

#### Main exposures (knowledge and attitude score)

Physician knowledge was measured using a 20-item scale to assess the diversity of knowledge required for effective notification of a range of conditions that may present in primary care settings. Each item had a single correct response with one point given for a correct answer and zero for an incorrect or unknown answer (Table 2). The total knowledge score was the sum of the 20-item scores, which was categorized for analysis purposes into poor/moderate knowledge (0–13 points) or good knowledge (14–20 points). Physician attitude was measured using five separate statements on a 5-point Likert scale (1 = strongly disagree to 5 = strongly agree), with a higher score indicating a more positive attitude. The item scores were summed to create a total attitude score, which was categorized into poor attitude (≤ 15 points) or good attitude (> 15 points) [12]. The categorical variables for physician knowledge and attitude were included in regression models as separate binary exposures.

#### Demographic and professional characteristics

Variables for key demographic and professional characteristics were included in analyses based on evidence of their relationship to the disease notification practices: age (30 years or younger, 31–40 years, 41 years or older) [13, 14]; gender (male, female) [14]; prior training on disease notification in the past 12 months (yes, no) [15, 16]; prior feedback from a supervisor regarding the diagnosis and notification of communicable diseases in the past 12 months (yes, no) [17]; and medical specialty group (general practitioner, family medicine, other specialties) [13]. Other medical specialties included pediatricians, gynecologists/obstetricians, dermatologists, and internal medicine physicians [18]. Categories for variables (e.g. age group, practice duration, and patient volume) were empirically defined based on data distributions together with analytical considerations of expected patient volumes in primary healthcare centres in West Bank, Palestine (see Table 1) [5].

#### Outcomes (notification practice)

All respondents were asked in separate questions if they encountered a suspected case of measles, mumps or chickenpox in their clinical practice within the past year, and if so, whether they notified the most recent case to authorities. Mumps, measles, and chickenpox notification were the three main outcomes in study analyses.

### Statistical analysis

We evaluated internal consistency of the knowledge and attitude scales using Cronbach’s alpha (knowledge α = 0.56, attitude α = 0.57). We also conducted item-level analysis for each item of the composite measure (Supplementary Table S1-S2). Convergent validity was assessed by examining correlations between knowledge and attitude scores using Spearman’s rank correlation coefficient, under the hypothesis that higher knowledge would be associated with more positive attitudes towards disease notification. Unweighted descriptive statistics were used to tabulate knowledge and attitude levels, demographic characteristics, and notification practices for the target population of physicians actively working in primary healthcare centers in the West Bank. We tested for associations between knowledge and attitude scores and physician demographics using Fisher’s Exact tests.

We used multivariable logistic regression models to quantify the associations between the main exposures (knowledge and attitude) and the three separate binary outcomes of measles, mumps, or chickenpox notification in the most recent encounter in the past 12 months. Standard logistic regression with HC3 robust standard errors was used for mumps and chickenpox outcomes. Since the measles model had a relatively limited number of observations, Firth penalized logistic regression was used to reduce potential small-sample bias. We initially estimated crude odds ratios (OR) with 95% confidence intervals (CI) for the association between the exposures and each outcome variable and subsequently adjusted the odds ratios for all variables listed in the methods section. Multi-collinearity was assessed using Variance Inflation Factors (VIF) prior to regression modelling. The level of statistical significance was set to 0.05. Data analysis was conducted in R (2023.06.0 Build 421).

### Ethical considerations

Participants were required to provide electronic informed consent before accessing and completing the online questionnaire. The study protocol was approved by the Scientific Research Review Board of the Primary Healthcare and General Health Directorate, Palestinian Ministry of Health (1037–41092; 5 December 2023).

## Results

### Sample characteristics

We asked 269 primary healthcare physicians to participate in the study and 223 completed the survey, corresponding to an 82.9% response rate. Among respondents, 58.7% were male with a median age of 35 years (IQR: 32–43 years). Three-quarters of respondents were general practitioners (74.9%) followed by family medicine specialists (13.5%), and other medical specialties (11.7%). Geographically, 39.9% of respondents worked in the southern region of the West Bank, while 37.7% worked in the northern region, and 22.4% in the middle region (Table 1).

Overall, 30.9% (*n* = 69) of physicians were categorized as having a good level of knowledge of the notification process with a mean score of 12.0 (SD 2.9, range 5–19), and 93.3% of physicians (*n* = 208) were categorized as having a good attitude towards the disease notification process (Table 1).

Significantly higher knowledge levels were observed among family medicine specialists and among physicians with more than 10 years of practice. Physicians in the northern region of the West Bank had more positive attitudes towards the Palestinian surveillance system than those in other regions (Table 1).

**Table 1:** Demographic characteristics of primary healthcare physicians in the West Bank in 2024–2025, by knowledge and attitude levels

Demographic Characteristics	Total	Knowledge			Attitude		
		Poor/Moderate	Good		Poor	Good	
	*n*	*n* (%)	*n* (%)	*p*-value^1^	*n* (%)	*n* (%)	*p*-value^1^
**Total**	223	154 (69.1)	69 (30.9)		15 (6.7)	208 (93.3)	
**Gender**				0.466			0.174
Male	131	93 (71.0)	38 (29.0)		6 (4.6)	125 (95.4)	
Female	92	61 (66.3)	31 (33.7)		9 (9.8)	83 (90.2)	
**Patients seen per day**				0.468			0.107
≤ 50 patients/day	111	79 (71.2)	32 (28.8)		11 (9.9)	100 (90.1)	
> 50 patients/day	107	71 (66.4)	36 (33.6)		4 (3.7)	103 (96.3)	
**Region** ^**2**^				0.094			0.047
Northern	84	52 (61.9)	32 (38.1)		7 (8.3)	77 (91.7)	
Middle	50	40 (80.0)	10 (20.0)		6 (12.0)	44 (88.0)	
Southern	89	62 (69.7)	27 (30.3)		2 (2.2)	87 (97.8)	
**Specialty Group**				0.004			0.590
General Practitioner	167	120 (71.9)	47 (28.1)		10 (6.0)	157 (94.0)	
Family Medicine	30	13 (43.3)	17 (56.7)		3 (10.0)	27 (90.0)	
Other^3^	26	21 (80.8)	5 (19.2)		2 (7.7)	24 (92.3)	
**Age Group**				0.777			0.235
≤ 30 years	45	32 (71.1)	13 (28.9)		3 (6.7)	42 (93.3)	
31–40 years	114	80 (70.2)	34 (29.8)		5 (4.4)	109 (95.6)	
> 40 years	64	42 (65.6)	22 (34.4)		7 (10.9)	57 (89.1)	
**Practice Duration**				0.038			0.383
≤ 10 years	145	107 (73.8)	38 (26.2)		8 (5.5)	137 (94.5)	
> 10 years	68	40 (58.8)	28 (41.2)		6 (8.8)	62 (91.2)	

^1^ Fisher’s Exact Test used to test for significance of association across the categorical variables. ^2^District regions: Northern (Qalqilya, Jenin, Tubas, Nablus, Tulkarm, Salfit), middle (Ramallah, Jerusalem, Jericho), southern (Dura, Yatta, Hebron, North Hebron, Bethlehem) ^3^Other specialties: Pediatrician (*n* = 14 (6.3%)), gynecologist/obstetrician (*n* = 7 (3.1%)), internal medicine physician (*n* = 1 (0.4%)), dermatologist (*n* = 1 (0.9%)), and other (*n* = 1 (0.9%))

### Knowledge, attitudes, and practice

Among the 223 respondents, 61.0% (*n* = 136) incorrectly believed that laboratory confirmation is required to notify a communicable disease. When presented with case scenarios, 67.3% (*n* = 150) correctly reported that a child who presents with sudden onset of weakness in both lower limbs is a notifiable case (Table 2).

With regards to surveillance case definitions, 34.5% (*n* = 77) of the 223 respondents reported that the surveillance case definition does not always consist of both clinical and laboratory criteria (Table 2). Only 17.0% (*n* = 38) correctly identified that symptom severity and laboratory results are not the basis for categorizing surveillance case definitions. Correct identification of notification timeframes was highest for diseases requiring immediate notification, including acute flaccid paralysis, rabies, and measles, with more than 80% of respondents answering correctly. In contrast, less than half of physicians answered correctly for diseases that require notification within a week or a month, including brucellosis, hepatitis A, hepatitis B, and mumps, as well as non-notifiable diseases such as shingles (Table 2).

**Table 2:** Variables included in the knowledge and attitude scores and the proportion of primary healthcare physicians (*n* = 223) in the West Bank in 2024–2025 with correct responses

Item	Correct Response	Correct *n* (%)
Knowledge^1^		
**Policies**		
Are you familiar with the Palestinian Preventive Medicine Department’s communicable disease policy?	Yes	191 (85.7)
Are you familiar with Palestinian Preventive Medicine Department’s official notification forms?	Yes	187 (83.9)
Is laboratory confirmation required prior to notification of a communicable disease?	No	87 (39.0)
**Which of these cases are notifiable?**		
Suspected rabies exposure following a dog bite	Notifiable	174 (78.0)
Cluster of suspected foodborne gastroenteritis cases	Notifiable	162 (72.6)
Suspected acute flaccid paralysis (AFP)	Notifiable	150 (67.3)
Asymptomatic hyperbilirubinemia	Not notifiable	168 (75.3)
**What is the notification timeframe for each of the following diseases (if notifiable)?** ^**2**^		
Bacterial pneumonia	Not required	153 (68.6)
Acute flaccid paralysis	Immediately	195 (87.4)
Mumps	Within a week	99 (44.4)
Measles	Immediately	181 (81.2)
Hepatitis A	Within a week	106 (47.5)
Hepatitis B	Within a week	102 (45.7)
Shingles	Not required	64 (28.7)
Rabies	Immediately	196 (87.9)
Chickenpox	Within a month	136 (61.0)
Brucellosis	Within a week	108 (48.4)
**Regarding case definitions**,** which of the following statements is correct?**		
Case definitions require both lab and clinical criteria	Incorrect	77 (34.5)
Surveillance case definitions are not intended for clinical diagnosis	Correct	100 (44.8)
Surveillance case definitions are categorized based on the severity of symptoms and laboratory results	Incorrect	38 (17.0)
**Attitude** ^**3**^		
Current notification system is convenient	Strongly Agree or Agree	123 (55.1)
Confidence in knowledge of disease notification protocols	Strongly Agree or Agree	106 (47.5)
Notification of communicable diseases is the physician’s responsibility	Strongly Agree or Agree	192 (86.1)
Notification of communicable diseases prompts action by relevant authorities	Strongly Agree or Agree	178 (79.8)
Knowledge of Palestinian Preventive Medicine Department’s policy is important	Strongly Agree or Agree	204 (91.4)

^1^Knowledge score: 20-item scale (1 = correct, 0 = incorrect, total score range: 0–20) ^2^Correct notification times were defined in accordance with the Palestinian national guidelines for disease notification. ^3^Attitude score: 5-point Likert scale (1 = strongly disagree to 5 = strongly agree; total score range: 5–25)

Among respondents who encountered a suspected case in the past year, the majority stated they notified their most recently encountered case of mumps (80.9%, *n* = 114/141), measles (80.8%, *n* = 63/78), or chickenpox (82.6%, *n* = 171/207). However, timely notification in accordance with national guidelines was substantially lower for mumps and chickenpox, where fewer than one in five physicians notified the most recent case within the recommended timeframe. In contrast, timely notification was higher for measles, for which 52.4% (*n* = 33) of 63 cases were notified immediately (Table 3).

In terms of general notification practices, 62.3% (*n* = 139) of respondents stated that doctors at their primary healthcare centers typically wait for laboratory confirmation to notify diseases, and only 34.1% (*n* = 76) attended an educational session for disease notification in the past year (Table 3).

**Table 3:** Proportion of primary healthcare physicians in the West Bank in 2024–2025 with correct practices

Item	*n* (%)	*N* ^1^
**Mumps notification**		
Encountered suspected mumps case in past year	141 (63.2)	223
Notified most recently encountered case	114 (80.9)	141
Notified most recently encountered case within the recommended timeframe (within a week)^2^	22 (19.3)	114
**Measles notification**		
Encountered suspected measles case in past year	78 (35.0)	223
Notified most recently encountered case	63 (80.8)	78
Notified most recently encountered case within the recommended timeframe (immediately)^2^	33 (52.4)	63
**Chickenpox notification**		
Encountered suspected chickenpox case in past year	207 (92.8)	223
Notified most recently encountered case	171 (82.6)	207
Notified most recently encountered case within the recommended timeframe (within a month)^2^	24 (14.0)	171
**Notification practices**		
Do not wait for laboratory confirmation before notification	84 (37.7)	223
Use official notification forms for notification	199 (89.2)	223
Attended disease notification training in the past year	76 (34.1)	223

^1^For conditional practice questions, N represents the subset eligible for the question ^2^Correct notification times were defined in accordance with the Palestinian national guidelines for disease notification

### Associations with disease notification practices

Crude and adjusted odds ratios are presented in Table 4, to show the association between KAP scores and notification behavior of primary healthcare physicians. In adjusted regression models, we found that knowledge and attitude categories were not significantly associated with notification of mumps, measles, or chickenpox during the most recent clinical encounter in the past year (Table 4). Other variables included in the adjusted regression models were associated with notification of these conditions. Compared with general practitioners, other medical specialties had significantly lower odds of notifying a mumps and chickenpox case although no association was found for measles notification. There were higher odds of notifying a suspected chickenpox case among physicians who had received supervisory feedback about disease diagnosis and notifications in the past year (Table 4).

**Table 4:** Factors associated with correct notification practices of mumps, measles, and chickenpox among primary healthcare physicians in the West Bank, 2024–2025

Predictor	Mumps			Measles			Chickenpox		
	*n* (%)^1^	Crude OR (95% CI)	Adjusted OR (95% CI)	*n* (%)^1^	Crude OR (95% CI)	Adjusted OR (95% CI)	*n* (%)^1^	Crude OR (95% CI)	Adjusted OR (95% CI)
**Knowledge**									
Poor/Moderate	71 (78.9%)	1.00	1.00	44 (77.2%)	1.00	1.00	111 (79.3%)	1.00	1.00
Good	43 (84.3%)	1.44 (0.60–3.75)	1.71 (0.54–5.45)	19 (90.5%)	2.81 (0.69–19.07)	2.33 (0.60–13.11)	60 (89.6%)	2.24 (0.97–5.83)	2.58 (0.92–7.18)
**Attitude**									
Poor	4 (57.1%)	1.00	1.00	4 (80.0%)	1.00	1.00	10 (90.9%)	1.00	1.00
Good	110 (82.1%)	3.44 (0.64–16.59)	2.13 (0.39–11.66)	59 (80.8%)	1.05 (0.05–7.84)	0.83 (0.07–5.99)	161 (82.1%)	0.46 (0.02–2.52)	0.36 (0.02–5.69)
**Gender**									
Male	77 (82.8%)	1.00	1.00	44 (80.0%)	1.00	1.00	101 (82.8%)	1.00	1.00
Female	37 (77.1%)	0.70 (0.30–1.69)	0.70 (0.23–2.18)	19 (82.6%)	1.19 (0.35–4.71)	1.39 (0.37–6.17)	70 (82.4%)	0.97 (0.47–2.04)	0.92 (0.37–2.31)
**Specialty**									
General Practitioner	92 (86.0%)	1.00	1.00	52 (81.3%)	1.00	1.00	133 (85.8%)	1.00	1.00
Family Medicine	13 (68.4%)	0.35 (0.12–1.13)	0.38 (0.08–1.70)	5 (83.3%)	1.15 (0.16–23.18)	0.77 (0.11–8.48)	24 (82.8%)	0.79 (0.29–2.55)	0.71 (0.20–2.56)
Other	9 (60.0%)	0.24 (0.08–0.82)	0.21 (0.06–0.81)	6 (75.0%)	0.69 (0.14–5.12)	0.78 (0.17–4.90)	14 (60.9%)	0.26 (0.10–0.68)	0.23 (0.08–0.69)
**Age**									
≤ 30 years	24 (82.8%)	1.26 (0.44–4.19)	1.01 (0.25–4.13)	10 (71.4%)	0.43 (0.10–1.95)	0.38 (0.08–1.75)	37 (88.1%)	1.92 (0.72–6.06)	1.88 (0.57–6.16)
31–40 years	61 (79.2%)	1.00	1.00	35 (85.4%)	1.00	1.00	85 (79.4%)	1.00	1.00
> 40 years	29 (82.9%)	1.27 (0.47–3.84)	1.18 (0.35–3.96)	18 (78.3%)	0.62 (0.16–2.40)	0.67 (0.18–2.66)	49 (84.5%)	1.41 (0.62–3.45)	1.72 (0.64–4.63)
**Prior Training**									
No training	68 (80.0%)	1.00	1.00	28 (77.8%)	1.00	1.00	109 (80.7%)	1.00	1.00
Training	46 (82.1%)	1.15 (0.49–2.82)	0.70 (0.23–2.14)	35 (83.3%)	1.43 (0.46–4.54)	1.58 (0.44–5.96)	62 (86.1%)	1.48 (0.69–3.40)	0.83 (0.29–2.39)
**Feedback received**									
No feedback	57 (76.0%)	1.00	1.00	25 (75.8%)	1.00	1.00	91 (76.5%)	1.00	1.00
Feedback	57 (86.4%)	2.00 (0.85–5.02)	2.32 (0.73–7.33)	38 (84.4%)	1.74 (0.56–5.54)	1.44 (0.42–5.03)	80 (90.9%)	3.08 (1.38–7.58)	4.01 (1.40–11.43)

^1^n (%) represents the number and percentage of physicians within each subgroup who reported notifying a case among those who encountered that disease in the past year (mumps: 114/141; measles: 63/78; chickenpox: 171/207). Multivariable logistic regression models separately quantified the association between physician knowledge scores and demographic characteristics with correct notification practices for each of the suspected diseases in the most recent clinical encounter within the past year. Measles model estimated using Firth’s penalized logistic regression. Measles multivariable model estimates should be interpreted with caution due to the limited sample size and wide confidence intervals. Note: District and practice duration not included due to model instability and multicollinearity

### Item-level Analysis

Corrected item-total correlations for the knowledge scale ranged from − 0.03 to 0.43, with the highest correlations observed for the chickenpox (0.43), mumps (0.39), hepatitis B (0.39), and brucellosis (0.39) notification timeframe items (Supplementary Table S1). Corrected item-total correlations for the attitude scale ranged from 0.25 to 0.45, with the highest correlation observed for the statement that notification of communicable diseases is the physician’s responsibility (0.45) (Supplementary Table S2). For both scales, deletion of individual items resulted in only minimal changes to Cronbach’s alpha. Spearman’s rank correlation showed a weak positive association between knowledge and attitude scores (ρ = 0.249, *p* < 0.001).

### Perceived barriers and preferences for disease notification

The most commonly reported barriers to disease notification were lack of time accompanied by large workloads (60.5%, *n* = 135), lack of knowledge about which diseases to notify (38.1%, *n* = 85) and when to notify the diseases (32.3%, *n* = 72) (Table 5). Only 41.7% (*n* = 93) of physicians reported receiving supervisory feedback on their diagnosis and notification practices for communicable diseases in the past year (Table 5). In open-ended responses about perceived barriers and recommendations, 8.5% (*n* = 19) of physicians suggested routine meetings and regular feedback could help improve the notification process.

The preferred method of notification among respondents was paper notification form (71.8%, *n* = 160) followed by phone call 54.7% (*n* = 122) (Table 5). Other responses in open-ended questions included suggestions for digital notification methods, such as WhatsApp and other app-based notification methods (2.7%, *n* = 6) or using the already integrated health information system (1.4%, *n* = 3). Two-thirds (69.5%, *n* = 155) of the physicians believed that they could benefit from learning more about the recognition of early signs of notifiable diseases, understanding the timing for disease notification (61.4%, *n* = 137), and gaining familiarity with the list of notifiable diseases (53.8%, *n* = 120) (Table 5).

**Table 5:** Perceived barriers and feedback on the disease notification system among primary healthcare physicians in the West Bank, 2024–2025. (*N* = 223)

Item	*n* (%)
**Perceived Barriers** ^1^	
Not enough time or too much workload	135 (60.5)
Uncertainty about which diseases to notify	85 (38.1)
Uncertainty about when to notify diseases	72 (32.3)
Low perception of the importance of disease notification	59 (26.5)
Do not know how or to whom to notify	38 (17.0)
Lack of confidence in health authorities taking it seriously	27 (12.1)
Concerns about violating patient trust	26 (11.7)
Lack of reward for notification	26 (11.7)
Lack of penalty for not notifying	25 (11.2)
Belief that others will notify	18 (8.1)
**Feedback**	
**Have you received any supervisory feedback on the diagnosis and notification of communicable diseases in the past 12 months (yes)**	93 (41.7)
**What methods do you prefer to use for disease notification** ^**1**^	
Paper notification form	160 (71.7)
Phone call	122 (54.7)
Mobile app-based notification	44 (19.7)
Email	23 (10.3)
Fax	10 (4.5)
Mobile SMS-based notification	9 (4.0)
**Which aspects of disease notification would you benefit from further training in** ^**1**^	
Recognizing early signs of notifiable diseases	155 (69.5)
Understanding the timing for disease notification	137 (61.4)
Familiarity with the list of diseases requiring notification	120 (53.8)
Proper methods of disease notification	91 (40.8)
**Which educational approach would you prefer for disease notification training** ^**1**^	
Workshops or seminars	162 (72.6)
Routine meetings	98 (43.9)
Interactive case studies or simulations	88 (39.5)
Printed educational materials	83 (37.2)
Online training modules	63 (28.3)
**How often would you like to participate in educational sessions on disease notification**	
Very often/Occasionally	196 (87.9)
Rarely/Not interested	27 (12.1)

^1^Respondents could select more than one response option for this item

## Discussion

Our study found that only one-third of physicians working in primary healthcare settings were categorized as having a good level of knowledge regarding the disease notification process in West Bank, Palestine. Despite low knowledge levels, over 80% of respondents reported notifying their last encountered measles, mumps, or chickenpox case. The knowledge score was designed to assess several operational domains of disease notification, ranging from familiarity with notification policies to disease-specific reporting requirements. Consequently, the composite score should be interpreted in the context of this broad construct, while the item-level findings provide additional insight into specific knowledge gaps for future training and system-level evaluation.

Existing literature has shown that improving knowledge of disease notification is an important first step towards improving notification practices [8, 13]. However, improving knowledge alone has often not been sufficient to improve practices in clinical settings due to the additional influences of social norms or working conditions such as workload and time constraints, perceived importance of notification by the respondent and their peers, supervisory oversight and feedback, and the ease of the notification process itself which corroborates with the barriers identified in this study [8, 10, 13–16, 18–21]. Consistent with such research, the adjusted analysis knowledge and attitude scores were not independently associated with notification behavior, although a borderline association was observed between knowledge and chickenpox reporting. On the other hand, receipt of supervisory feedback was associated with higher self-reported notification rates for chickenpox. The lack of association between knowledge scores and notification practices in our study may also reflect methodological limitations and should be interpreted with caution. Notification practices were based on physicians’ self-report of the most recent suspected case encountered during the previous year, which makes it plausible that recall and social desirability bias could have obscured the true relationship [22–25]. Despite these limitations, our findings suggest that interventions aiming to strengthen notification practices should extend beyond knowledge-based training to address organizational barriers as well as motivational and reinforcement mechanisms such as feedback.

Low levels of knowledge among healthcare workers in general, and physicians in particular, has been a long-standing challenge to mandatory notifications in different settings [26]. Studies from Jordan [8], Ireland [19], and the West Bank [6] found insufficient knowledge about which diseases to notify and how to notify them as a main cause for underreporting in surveillance systems. These studies also pointed to the need for building staff capacity in the notification process by strengthening awareness and knowledge of the system. In our study, a substantial proportion of physicians reported a lack of knowledge regarding which diseases to notify and when to notify them, which has been documented as barriers in other studies in both high- and low-income countries [7–11, 20, 21, 27]. This highlights the need for easily accessible reference materials that physicians can refer to during routine clinical practice when uncertainty arises, alongside continuous education and supportive supervision on notification guidelines.

There appeared to be a mismatch between physicians’ knowledge of disease notification and the high notification rates reported for their most recent clinical encounters. This raises the possibility that reported practices are not an accurate reflection of actual notification behaviour which could, as noted earlier, be partly due to recall or social desirability bias [22–25], although notification rates to the Palestinian Preventive Medicine Department remain unknown limiting comparisons with our self-reported findings. Moreover, the pattern of high notification rates appeared consistent across physicians from different medical specialties for measles, whereas for diseases that are perceived as more common and less severe (e.g., mumps and chickenpox) the difference in reported notifications between specialties was more pronounced, as reflected in the adjusted analysis. This could indicate underreporting for common and less severe conditions by certain physician specialities, even if notification is mandatory, requiring enhanced training and awareness of guidelines [10, 16].

The high reported notification rates failed to translate into timely notification. The majority of respondents reported notifying the last case of mumps, measles, or chickenpox they encountered. However, only half of notified measles cases were reported immediately, while fewer than one in five notified mumps and chickenpox cases were reported within the recommended notification timeframes. These findings should also be interpreted within the context of the challenges facing the health system in the West Bank, as the study was conducted during a period of deteriorating security conditions. Increased movement restrictions and pressures on the healthcare system might have affected physician workload and routine disease notification practices [28, 29].

Among the barriers identified in this study, a few are particularly noteworthy due to their commonality and direct relevance to notification timeliness. Misconceptions regarding laboratory confirmation requirements were common. Two-thirds of physicians believed that surveillance case definitions must always consist of both clinical and laboratory criteria, and a similarly high proportion believed that laboratory confirmation is required before notification. As such, providing physicians with clear guidelines to reinforce clinical suspicion as sufficient grounds for notifying, without waiting for laboratory confirmation, could potentially help improve notification timeliness. Delays related to laboratory confirmation have been frequently identified in literature as a major cause of reducing timeliness and completeness of disease notification in infectious disease surveillance systems [11, 26, 30]. In the West Bank context, laboratory delays have been documented as a barrier to timely disease notification [31].

Lack of time and excessive workload were the most frequently reported barriers overall, and as discussed above, this might have been exacerbated by the operational challenges facing the West Bank health system during the study period. The current system uses paper-based notification and diagnostic samples often must be sent from local clinics to central reference laboratories for testing, all of which is time-consuming and is likely to delay notification [6]. Addressing these barriers would require simplifying the notification procedures and reducing the time required to complete notifications through more efficient electronic notification methods, alongside efforts to improve physicians’ knowledge of the notification system. Although most physicians expressed a preference for paper-based notification, open-ended responses in our survey also identified an emerging interest for digital notification tools, suggesting a potential shift to alternative notification methods that are easier to use and that have improved notification systems in other settings [32, 33]. Issues with timely notification of communicable diseases are a well-known problem in many settings due to lack of time, excessive workloads, and common misconceptions as found in our study [8, 10, 19, 21].

Nearly all physicians reported a positive attitude towards the disease notification system, implying a willingness to engage in the system and potential to build capacity and knowledge. However, only one-third of respondents attended any training on the notification system in the past year, highlighting a missed opportunity to provide regular training on notification requirements and guidelines, and maintain familiarity with notification procedures over time. Furthermore, our findings suggest that feedback mechanisms could play a supportive role in notification practices as many physicians emphasized the need for a functional feedback mechanism, which is consistent with previous research [27, 34, 35]. Together with the higher self-reported notification rates observed among physicians who received supervisory feedback, these findings underscore the need to reinforce feedback loops within the notification system.

The moderate internal consistency observed for the knowledge and attitude scales should be interpreted in the context of the constructs they were intended to measure [36, 37]. The knowledge scale assessed several operational domains of disease notification, given the wide-ranging and distinct areas of knowledge required by primary care physicians for the notification system. Consequently, strong correlations between all items were not necessarily expected, as physicians may be familiar with certain aspects of the notification process while having limited knowledge of others [37]. Item-level analysis provided further insight into the performance of the knowledge and attitude scales. Several knowledge items, including those assessing rabies and measles notification timeframes, surveillance case definition categorization, and bacterial pneumonia reporting requirements, demonstrated near-zero or negative corrected item-total correlations. These items also showed highly skewed response distributions, with most respondents answering either correctly or incorrectly. In contrast, items assessing chickenpox, mumps, hepatitis B, and brucellosis notification timeframes demonstrated the highest corrected item-total correlations and more balanced response distributions. Corrected item-total correlations for the attitude scale were consistently positive across all items, suggesting more consistent performance across the individual attitude items. Furthermore, deletion of individual items resulted in minimal changes in Cronbach’s alpha for both scales, indicating that no single item substantially influenced the overall internal consistency of either scale.

There are some methodological limitations in this study. First, while the sample was sufficient to draw overall conclusions, certain subgroups such as the number of notified measles cases were relatively small such that statistical power was reduced and confidence intervals were wide. Moreover, confidence intervals for attitudes were particularly wide across all three notification outcomes, likely reflecting the limited variability in attitude scores, with nearly all physicians categorized as having positive attitudes. This ceiling effect may have reduced the precision of the estimated associations and limited the ability to detect statistically significant relationships. Therefore, findings from the measles regression model and the attitude-related associations should be interpreted with caution. Furthermore, the moderate Cronbach’s alpha values observed for the knowledge and attitude scales suggest item heterogeneity. As a result, the use of composite scores may have obscured variability across individual items and masked differences in specific knowledge or attitude components related to disease notification, as shown in the item-level analysis. It is also important to note that although our data indicates knowledge gaps and practices consistent with under- or delayed notification, self-reported practices as used in this study are not direct measurements. Second, social desirability bias may lead respondents to provide answers that may be viewed as acceptable to the research team, especially in relation to sensitive non-compliance questions. The self-administered online questionnaire used in this study may partially reduce this bias [22, 24, 25]. Recall bias could also be an issue for reporting on clinical practices up to one year later in a survey setting [38], which could potentially lead to higher reports. Third, observational studies are prone to residual confounding making causal inferences difficult. Temporal associations needed for causal inferences are further obscured in this cross-sectional survey since knowledge and attitudes reflect respondent recall on the survey date while reported behaviours reflect notification practices up to one year earlier. Finally, the study sampled physicians working in the Palestinian Ministry of Health primary healthcare centers in the West Bank, and results may not be generalizable to physicians in the private sector, non-governmental organizations, or hospital settings.

## Conclusion

In conclusion, while primary healthcare physicians in the West Bank demonstrated a willingness to notify diseases, we found that knowledge gaps and misconceptions coexisted with delayed self-reported notification that represent key targets for future training and system-level evaluation. Findings further highlight the need for targeted training to all medical specialties who are engaged in the notification process with a focus on notification criteria and timing, dissemination of clear notification guidance, and implementation of a feedback mechanism. Addressing the reported barriers and preferences for notifying diseases found in our study could further improve the performance of the disease notification system.

## Supplementary Information

Below is the link to the electronic supplementary material.

Supplementary Material 1

## Data Availability

The datasets generated and analyzed during the current study are not publicly available due to confidentiality and ethical restrictions. The study questionnaire and datasets are available from the corresponding author upon reasonable request.
