# Supplementary material for: Barriers to disease notification among primary healthcare physicians in West Bank, Palestine: a cross-sectional survey
Source: BMC Health Serv Res. 2026 Aug 10;26:1554. doi: 10.1186/s12913-026-15297-1 (PMC13746514; doi:10.1186/s12913-026-15297-1)
Supplement: Supplementary file 1 [file 12913_2026_15297_MOESM1_ESM.docx]

Supplementary Table S1: Item-level Analysis of knowledge items.

| **Item** | **Corrected Item-Total Correlation** | **Alpha if Item Deleted** |
| --- | --- | --- |
| **Policies** |  |  |
| Are you familiar with the Palestinian Preventive Medicine Department's communicable disease policy? | 0.14 | 0.56 |
| Are you familiar with Palestinian Preventive Medicine Department’s official notification forms? | 0.20 | 0.55 |
| Is laboratory confirmation required prior to notification of a communicable disease? | 0.17 | 0.55 |
| **Which of these cases are notifiable?** |  |  |
| Suspected rabies exposure following a dog bite | 0.03 | 0.57 |
| Cluster of suspected foodborne gastroenteritis cases | 0.14 | 0.56 |
| Suspected acute flaccid paralysis (AFP) | 0.34 | 0.53 |
| Asymptomatic hyperbilirubinemia | 0.10 | 0.56 |
| **What is the notification timeframe for each of the following diseases (if notifiable)?^2^** |  |  |
| Bacterial pneumonia | 0.09 | 0.57 |
| Acute flaccid paralysis | 0.22 | 0.55 |
| Mumps | 0.39 | 0.52 |
| Measles | -0.03 | 0.58 |
| Hepatitis A | 0.33 | 0.52 |
| Hepatitis B | 0.39 | 0.52 |
| Shingles | 0.03 | 0.58 |
| Rabies | -0.01 | 0.57 |
| Chickenpox | 0.43 | 0.51 |
| Brucellosis | 0.39 | 0.52 |
| **Regarding case definitions, which of the following statements is correct?** |  |  |
| Case definitions require both lab and clinical criteria | 0.17 | 0.55 |
| Surveillance case definitions are not intended for clinical diagnosis | 0.01 | 0.58 |
| Surveillance case definitions are categorized based on the severity of symptoms and laboratory results | 0.05 | 0.57 |

Supplementary Table S2: Item-level analysis of attitude items.

| **Item** | **Corrected Item-Total Correlation** | **Alpha if Item Deleted** |
| --- | --- | --- |
| Current notification system is convenient | 0.25 | 0.56 |
| Confidence in knowledge of disease notification protocols | 0.27 | 0.56 |
| Notification of communicable diseases is the physician’s responsibility | 0.45 | 0.45 |
| Notification of communicable diseases prompts action by relevant authorities | 0.36 | 0.49 |
| Knowledge of Palestinian Preventive Medicine Department’s policy is important | 0.34 | 0.51 |
